# Selection and Evaluation of Native Plants for Rain Gardens in Tropical Regions: A Dual‐Method Assessment Framework

**DOI:** 10.1002/pei3.70088

**Published:** 2025-10-03

**Authors:** Pei‐Chun Chen, Meng‐Yuan Huang, Shen‐Yong Wang

**Affiliations:** ^1^ Department of Landscape Architecture National Chiayi University Chiayi City Taiwan (ROC); ^2^ Department of Life Sciences National Chung Hsing University Taichung City Taiwan (ROC)

**Keywords:** chlorophyll fluorescence, plant selection framework, plant stress, rain gardens, tropical herbaceous species, urban green infrastructure, visual impairment assessment

## Abstract

Rain gardens depend on resilient plant species that can withstand fluctuating moisture conditions while providing ecological and aesthetic benefits. This study addresses the limited research on tropical and native herbaceous species by evaluating their health through both visual and physiological assessment methods. A pretest list of 44 species was developed through expert interviews, and plant responses were assessed using chlorophyll fluorescence, expressed as the maximum quantum yield of photosystem II (*F*_v_/*F*_m_), and visual damage ratings after a 15‐day continuous immersion experiment. Cluster analysis identified 24 immersion‐tolerant species, among which 10 native perennial herbaceous plants were selected for further testing. These species were subjected to repeated water stress cycles consisting of 7 and 14 days of immersion followed by 7 days of drought, repeated over three immersion and two drought intervals. Results indicated that nine species—*Aster indicus*, *Aster shimadae*, *Lobelia chinensis* , *Dianthus seisuimontanus*, *Dianthus superbus* , *Evolvulus alsinoides* , *Euphorbia formosana* , *Lespedeza cuneata* , and *Richardia scabra* —consistently maintained *F*_v_/*F*_m_ values above 0.7 throughout both flooding durations, reflecting high photosynthetic stability, indicating that they can tolerate such fluctuations in water availability. By contrast, *Eupatorium lindleyanum* exhibited *F*_v_/*F*_m_ above 0.7 in 66% and 33% of observations in the 7‐ and 14‐day treatments, respectively. These results provide insight into the selection of resilient native species and support the establishment of a standardized methodology for plant health assessment in rain garden design.

## 1 Introduction

Rain gardens, also known as bioretention systems, are one of the low‐impact development methods used in urban areas to address the problem of increased stormwater runoff due to urban development (Dietz and Clausen 2005; Hatt et al. 2009; Tang et al. 2016; Sharma and Malaviya 2021; Bąk and Barjenbruch 2022). They primarily use retention, infiltration, and evapotranspiration to reduce stormwater runoff, replenish groundwater, and increase biodiversity, and studies have shown that rain gardens can effectively reduce runoff and decrease nonpoint source pollution. In addition, vegetation in rain gardens plays a crucial role. It not only helps stabilize soil and filter pollutants (Muthanna et al. 2008; Osheen and Singh 2019; Kasprzyk et al. 2022), but also, as a component of the urban blue‐green interface, enhances overall landscape aesthetics and creates a pleasant environment (Davis et al. 2006; Muthanna et al. 2008; Read et al. 2008; Tang et al. 2016; Osheen and Singh 2019; Shi et al. 2024).

**FIGURE 1:**
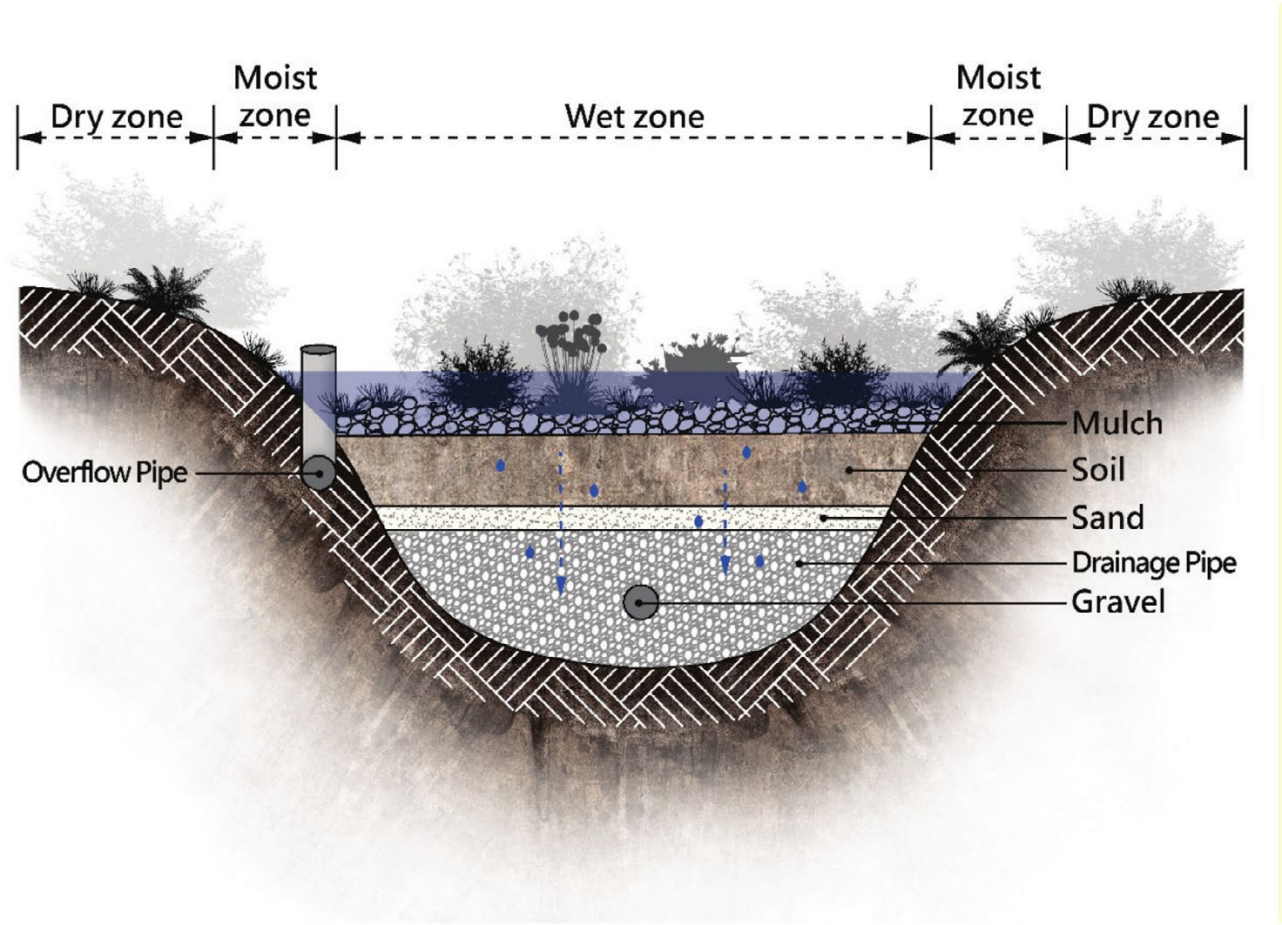
Rain garden cross‐section.

It is usually recommended to use native species when selecting plants for rain gardens (Dunnett and Clayden 2007; Ignatieva et al. 2008; Kasprzyk et al. 2022; Chaves et al. 2025), but because the microclimate in urban environments is usually hotter, the original soil has been changed, and native plants are less immune to pests, non‐native plants can also be used (Bortolini and Zanin 2019). Rainwater storage in rain gardens is achieved by maximizing infiltration and avoiding standing water (Dietz and Clausen 2005; Roy‐Poirier et al. 2010). At the same time, this creates limitations in the selection of rain garden plants. Changes in soil moisture can be significant, with lower areas being wetter and outer areas relatively drier (Figure 1), and during dry periods between rainy days, the good drainage of the soil also exacerbates the soil's dry conditions (Kasprzyk et al. 2022; Shi et al. 2024; Yuan and Dunnett 2018). Therefore, plants suitable for rain gardens need to be both drought‐resistant and able to withstand short‐term immersion (Nelson et al. 2018; Bortolini and Zanin 2019; Osheen and Singh 2019). The visual pleasure of outdoor spaces is one of the important issues in the field of landscape research, and plants are considered the most important component and influencing factor affecting public perceptions of landscapes (Ulrich 1981; Yılmaz et al. 2018). Perennial plants have significant diversity in their tolerance to immersion conditions, so selecting appropriate vegetation types and plants for use in rain gardens is not an easy task, and the use of inappropriate species may lead to planting failures or even unpleasant visual effects (Yuan and Dunnett 2018).

Traditionally, plant health has been assessed through visual observation, including assessments of leaf color, turgor, morphology, and the presence of disease or damage. This approach is widely used in landscape horticulture due to its simplicity and direct relevance to aesthetic goals (Contreras et al. 2013). However, visual symptoms of stress often emerge only after significant physiological deterioration has occurred, limiting their value as early warning indicators (Katuwal et al. 2023). In contrast, physiological measurements offer quantitative and often early indicators of plant stress. Parameters such as chlorophyll content, stomatal conductance, photosynthetic rate, and chlorophyll fluorescence (*F*_v_/*F*_m_) provide insight into plant water status, nutrient efficiency, and photosynthetic performance (Smethurst and Shabala 2003; Yuan and Dunnett 2018). These metrics have been used in controlled experiments to predict plant performance in fluctuating water conditions typical of rain gardens. Despite the growing availability of physiological tools and data, comparative research on the effectiveness, practicality, and complementarity of visual and physiological assessments remains limited, especially in the context of green infrastructure.

Several studies have investigated plant species suitable for rain gardens, such as Dunnett and Clayden (2007), Laukli et al. (2022), and Nelson et al. (2018). However, these experiments were mostly conducted in temperate regions of Europe, and their results primarily reflect plant adaptability under temperate climatic conditions. In contrast, studies on the suitability of native plant species in tropical and subtropical regions remain limited, resulting in scarce application references. Regarding the use of native versus non‐native species, previous studies have suggested that native plants are generally preferred for rain gardens due to their local adaptability and ecological compatibility (Osheen and Singh 2019; Yuan and Dunnett 2018), yet no clear consensus has been reached in the literature. These knowledge gaps restrict the ability to make evidence‐based design and management decisions for rain gardens under varying climatic conditions. Although more tools have been proposed in recent years, comparative research on their effectiveness, practicality, and complementarity in real‐world green infrastructure settings remains scarce. Therefore, this study aims both to identify plant species capable of tolerating environmental fluctuations and to develop a health assessment framework combining visual and physiological indicators, addressing the above research gaps.

## 2 Materials and Methods

### 2.1 Site and Materials

#### 2.1.1 Site and Dates

The experimental location was an unshaded and well‐ventilated site in Chiayi City, southwestern Taiwan. A pretest was conducted from March 9 to 24, 2021, for a total of 16 days, during which plants were continuously immersed in water. The average temperature was 22°C, with a maximum of 32°C, a minimum of 13°C, and an average relative humidity of 73%. The formal experiment, designed to evaluate plant tolerance to fluctuating moisture conditions (alternating immersion and drying cycles), was conducted from November 8, 2021, to January 24, 2022. During this period, the average temperature was 20°C, with a maximum of 31°C, a minimum of 13°C, and an average relative humidity of 76%.

#### 2.1.2 Cultivation Substrate

Nelson et al. (2018) suggested selecting a medium that induces the most adverse conditions in plant tolerance tests for immersion and drought. This allows the cultivation medium to quickly transition from a moist to a dry state, providing more adverse environmental conditions for plants. In this study, the cultivation medium was based on the construction of rain gardens and the research of Emanuel et al. (2010). The composition ratio of the medium was designed to increase the proportion of coarse sand with a relatively fast drainage rate, while avoiding rapid changes in soil moisture dry points, due to the large temperature difference between day and night and the short period of heavy rainfall in the experimental location. Therefore, a composition ratio of 60% coarse sand, 20% humus, and 20% topsoil was adopted as the cultivation medium for this study.

#### 2.1.3 Cultivation Containers

Yuan and Dunnett (2018) conducted an experiment to test the tolerance of 15 perennial species to periodic flooding, using 2 L permeable pots with an impermeable outer pot as the soaking container. In this study, considering that the production and engineering specifications of perennial herbaceous plants in Taiwan are mostly 3.5–6 in. (0.37–3 L) pots, 5.5 in. (1.73 L) pots were selected for the experimental plants. Periodic soaking and drought experiments were conducted beginning 20 days after the plants were transplanted into 5.5 in. pots (pot specifications: diameter 14 cm, height 13 cm, capacity about 1.73 L).

### 2.2 Establishment of the Experimental Plant List

#### 2.2.1 Expert Interviews

Three interviewees were involved in the study, including a plant ecologist with extensive knowledge of plant habitat requirements, and two landscape plant producers with 30 years of experience in plant propagation and cultivation, who regularly advise landscape companies on plant selection. The research team then evaluated this list and selected 44 species from 22 families of perennial herbaceous plants for use in the subsequent experimental study (Table 1), among them, 34 species are native, and the remaining 10 are either cultivars or naturalized species; all are readily available in the nursery market. The definition of native species follows the Catalogue of Life in Taiwan (TaiCOL), whereas tropical species were identified based on distribution records in Chiou et al. (2004) and species observation points from iNaturalist, where species distributed in tropical and subtropical regions were designated as tropical species.

**TABLE 1:** Values of visual assessments, chlorophyll fluorescence (*F*_v_/*F*_m_), and the water‐holding capacity before, during, and after immersion in the preliminary wet tolerance test.

Family name	Scientific name	Native	Tropical	Habitat	d0		d8		d15		Water‐holding capacity
					Visual assessment	*F*_v_/*F*_m_	Visual assessment	*F*_v_/*F*_m_	Visual assessment	*F*_v_/*F*_m_	
Nephrolepidaceae	*Nephrolepis auriculata*	✓	✓	Montane open area, Coastal sandy area	1	0.70	1	0.74	1	0.61	Tolerant
Polypodiaceae	*Phymatodes scolopendria*	✓	✓	Montane valley, Coastal gravel beach	1	0.84	1	0.78	1	0.80	Tolerant
Amaryllidaceae	*Zephyranthes candida*			Cultivar	1	0.83	1	0.82	1	0.82	Tolerant
Araceae	*Homalomena philippinensis*	✓	✓	Forest understory (moist type)	1	0.77	1	0.81	1	0.80	Tolerant
Asparagaceae	*Aspidistra elatior*	✓	✓	Montane open area	1	0.63	1	0.69	1	0.67	Tolerant
Asparagaceae	*Aspidistra elatior* ‘Punctata’			Cultivar	1	0.52	1	0.39	1	0.33	Tolerant
Asparagaceae	*Aspidistra elatior* ‘Variegata’			Cultivar	1	0.74	1	0.71	1	0.59	Tolerant
Asteraceae	*Aster indicus*	✓	✓	Moist open area	1	0.84	1	0.81	1	0.83	Tolerant
Asteraceae	*Aster shimadae*	✓		Low mountain and hill	1	0.81	1	0.84	1	0.84	Tolerant
Asteraceae	*Eupatorium clematideum* var. *gracillimum*	✓	✓	Montane open area	1	0.67	1	0.79	0.7	0.73	Intolerant
Asteraceae	*Eupatorium formosanum*	✓	✓	Montane open area, Grassland	1	0.83	0	0.02	0	0.00	Intolerant
Asteraceae	*Eupatorium lindleyanum*	✓		Low mountain and hill	1	0.81	1	0.80	1	0.77	Tolerant
Asteraceae	*Eupatorium clematideum*		✓	Montane open area	1	0.72	0	0.00	0	0.00	Moderately
Asteraceae	*Eupatorium hualienense*	✓		Coastal	1	0.76	0	0.00	0	0.00	Intolerant
Asteraceae	*Farfugium japonicum*	✓	✓	Montane open area	1	0.84	0.5	0.82	0.5	0.81	Moderately
Asteraceae	*Farfugium japonicum* var. *formosanum*	✓	✓	Montane open area	1	0.85	0.3	0.83	0.3	0.84	Moderately
Asteraceae	*Vernonia maritima*	✓	✓	Coastal	1	0.82	0.5	0.70	0.3	0.73	Moderately
Begoniaceae	*Begonia fenicis*	✓	✓	Forest edge, Coral reef area	1	0.81	1	0.68	0.7	0.75	Moderately
Campanulaceae	*Lobelia chinensis*	✓	✓	Lowland wetland	1	0.85	1	0.84	1	0.83	Tolerant
Caprifoliaceae	*Sambucus formosana*	✓	✓	Montane open area	1	0.84	1	0.82	1	0.76	Tolerant
Caryophyllaceae	*Dianthus palinensis*	✓		Montane open area	1	0.82	1	0.73	0.5	0.64	Moderately
Caryophyllaceae	*Dianthus seisuimontanus*	✓		Limestone area	1	0.78	1	0.83	1	0.85	Tolerant
Caryophyllaceae	*Dianthus superbus*	✓	✓	Montane open area	1	0.82	1	0.82	1	0.82	Tolerant
Caryophyllaceae	*Silene fortunei*	✓	✓	Montane open area, Coastal	1	0.81	0.5	0.75	0.5	0.51	Moderately
Chlopanthaceae	*Chloranthus spicatus*		✓	Montane open area	1	0.76	0.5	0.65	0.3	0.67	Moderately
Convolvulaceae	*Evolvulus alsinoides*	✓	✓	Coastal	1	0.75	1	0.80	1	0.74	Tolerant
Euphorbiaceae	*Euphorbia formosana*	✓	✓	Grassland	1	0.77	0.8	0.78	1	0.80	Tolerant
Gramineae	*Pennisetum setaceum*		✓	Montane open area, Grassland	1	0.84	1	0.86	1	0.87	Tolerant
Gramineae	*Pennisetum alopecuroides*		✓	Montane open area, Grassland	1	0.79	0.5	0.78	0.5	0.78	Moderately
Gramineae	*Pogonatherum crinitum*	✓	✓	Montane open area, Grassland, Moist area	1	0.75	1	0.74	0.7	0.79	Moderately
Lamiaceae	*Glechoma hederacea*		✓	Montane open area, Grassland, Moist area	1	0.82	1	0.82	0.7	0.74	Moderately
Lamiaceae	*Scutellaria barbata*	✓	✓	Lowland wetland	1	0.73	0.7	0.75	0.3	0.73	Moderately
Lamiaceae	*Scutellaria indica*	✓	✓	Montane open area, Grassland	1	0.79	0.5	0.66	0.3	0.66	Moderately
Lamiaceae	*Scutellaria playfairi*	✓	✓	Montane open area	1	0.78	0.7	0.55	0.3	0.56	Moderately
Lamiaceae	*Scutellaria tashiroi*	✓	✓	Montane open area	1	0.79	0.7	0.22	0	0.00	Moderately
Leguminosae	*Desmodium triflorum*	✓	✓	Montane open area, Grassland	1	0.74	1	0.77	1	0.73	Tolerant
Leguminosae	*Lespedeza cuneata*	✓	✓	Montane open area, Grassland	1	0.82	1	0.83	1	0.82	Tolerant
Orchidaceae	*Calanthe triplicata*	✓	✓	Forest understory	1	0.70	1	0.64	0.7	0.64	Moderately
Plantaginaceae	*Plantago asiatica*	✓	✓	Lowland plain	1	0.74	1	0.65	1	0.73	Tolerant
Polygonaceae	*Polygonum capitatum*		✓	Lowland wetland	1	0.84	1	0.83	1	0.60	Tolerant
Scrophulariaceae	*Mazus fauriei*	✓	✓	Lowland wetland	1	0.82	1	0.80	1	0.79	Tolerant
Umbelliferae	*Centella asiatica*	✓	✓	Lowland wetland	1	0.81	1	0.55	1	0.82	Tolerant
Verbenaceae	*Caryopteris incana*	✓	✓	Lowland plain, Grassland	1	0.80	1	0.81	0.7	0.76	Moderately
Verbenaceae	*Richardia scabra*		✓	Lowland wetland	1	0.74	1	0.84	1	0.83	Tolerant

*Note:* Water‐holding capacity is classified into three levels: tolerant, moderately (moderately tolerant), and intolerant.

Abbreviations: d0 = before immersion; d8 = eighth day of immersion; d15 = fifteenth day of immersion.

#### 2.2.2 15‐Day Immersion Pretest for Moisture Resistance

In most urban areas of Taiwan, the rainy season occurs from May to September, and the number of days with surface water in rain gardens often exceeds the recommended 3 days without standing water. Therefore, in the preliminary test of this study, 44 plant species obtained from interviews were screened for their tolerance to prolonged wet conditions. The evaluation methods included visual assessment and chlorophyll fluorescence (*F*_v_/*F*_m_) measurement. Visual assessment followed Nelson et al. (2018) and was classified into five levels based on the extent of plant damage: 1 = no damage (100%–80%), 0.7 = leaf wilting began (80%–60%), 0.5 = more than 60% of the plant wilted (60%–40%), 0.3 = leaf softening and necrosis began (40%–20%), 0 = plant death (20%–0%), with one sample per species for visual assessment. Chlorophyll fluorescence measurements were used to assess photosynthetic activity and plant health, with the *F*_v_/*F*_m_ ratio indicating the degree of photoinhibition. For each plant, three leaves marked with colored tape were measured (sample size = 3) using a WALZ MONITORING‐PAM chlorophyll fluorescence analyzer. After 15 days of immersion, the 44 perennial herbaceous plants were evaluated, and a two‐stage cluster analysis grouped them into three clusters. Subsequently, a *k*‐means analysis classified the plants into an intolerant immersion group (3 species), a moderately tolerant group (17 species), and a tolerant group (24 species) according to their tolerance levels (Table 1 and Figure 3).

#### 2.2.3 Plants Selection for Formal Experiment Assessing Tolerance to Cyclical Water Variation

Based on the results of cluster analysis, 10 native perennial herbaceous species were selected from the 24 species in the immersion‐tolerant group (Figure 3), which had endured 15 consecutive days of water immersion and showed higher average values for the chlorophyll fluorescence (*F*_v_/*F*_m_) ratio. These 10 species were then subjected to a formal experiment to evaluate their tolerance to cyclical water variation. Although the preliminary results indicated that better performance was not limited to native species, which is consistent with previous studies showing that horticultural cultivars can also be used, 79% of the immersion‐tolerant species were native, and many exhibited favorable performance. In addition, previous studies have emphasized the importance of selecting native plants for rain gardens due to their local adaptability and ecological compatibility (Osheen and Singh 2019; Yuan and Dunnett 2018). Considering that research on the use of native plants in rain gardens remains limited in Taiwan, native species were prioritized for the formal experiment. These 10 species, chosen as experimental plants, were *Aster indicus*, *Aster shimadae*, *Eupatorium lindleyanum*, *Lobelia chinensis* , *Dianthus seisuimontanus*, *Dianthus superbus* , *Evolvulus alsinoides* , *Euphorbia formosana* , *Lespedeza cuneata* , and *Richardia scabra* .

### 2.3 Simulation of Cyclic Soil Immersion

#### 2.3.1 Duration of Drought and Immersion Stress

Prior to formal measurements, preliminary tests were conducted to determine the stress tolerance of the 10 selected plant species. For drought, soil moisture and plant visual appearance were monitored continuously over several days without watering. A 7‐day period was selected as the drought stress duration, based on the point at which plants survived after rewatering. For immersion, pretests showed that plants in both the immersion‐tolerant and moderately tolerant groups did not exhibit significant declines in visual assessment or chlorophyll fluorescence before the 5th day of continuous submersion. Accordingly, two periods of immersion, namely 7‐ and 14‐day of continuous immersion, were adopted to impose more severe stress conditions.

#### 2.3.2 Timeline of Immersion and Drought Periods

**FIGURE 2:**
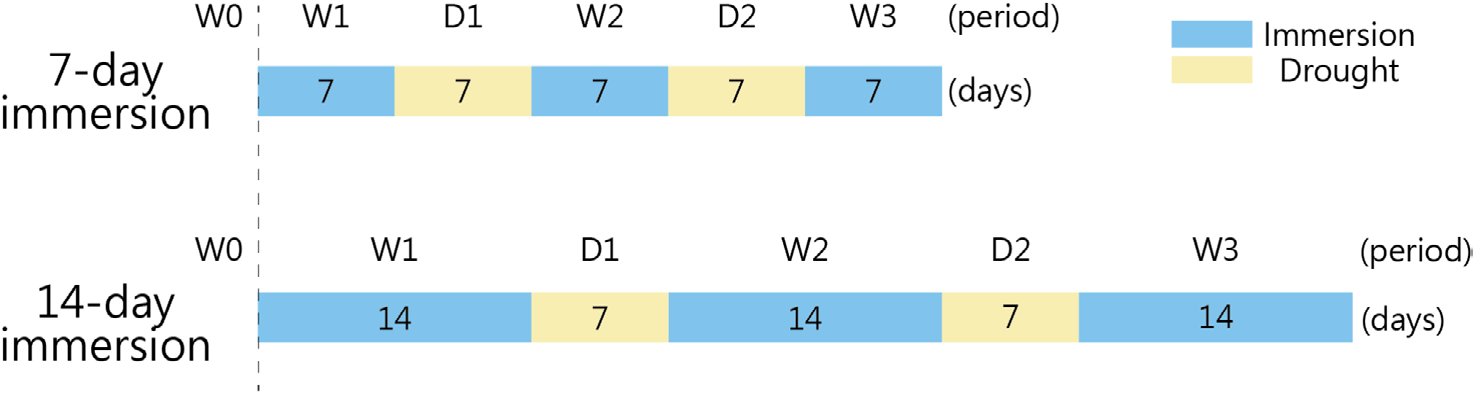
Experimental schedule for immersion and drought treatments.

The experiment included two immersion periods (7 and 14 days) and a 7‐day drought period. The cyclical water variation test involved three immersion events and two drought periods (Figure 2). Six measurement points were defined as follows: W0 = before immersion; W1 = first wet period; D1 = first dry period; W2 = second wet period; D2 = second dry period; W3 = third wet period.

### 2.4 Assessment of Plant Tolerance to Periodic Water Fluctuation Stress

In the pretest experiment, some plants showed minimal visual changes even after several consecutive days of immersion. After 15 days, 68% of the plants were evaluated as no damage or leaf wilting began, indicating negligible visual stress symptoms. Therefore, chlorophyll fluorescence was used in the formal experiment to assess physiological responses to periodic water fluctuation. Both experimental and control groups included three pots per species. In the experimental group, three leaves per pot were marked with colored tape and measured, resulting in a total of nine leaves per species for chlorophyll fluorescence analysis.

## 3 Results

### 3.1 Changes in Visual Assessment Indices and Chlorophyll Fluorescence (*F*_v_/*F*_m_) of 44 Pretest Plant Species After 15 Days of Continuous Immersion

**FIGURE 3:**
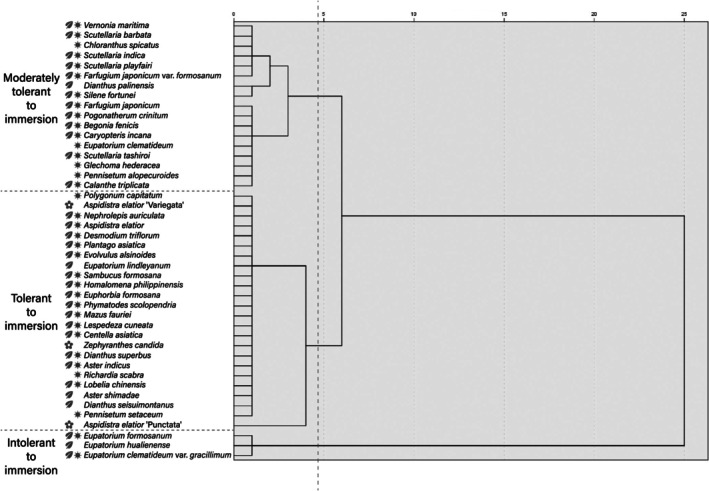
Dendrogram of the hierarchical cluster analytical results. 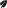: native plant; 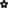: horticultural cultivar; 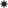: tropical plant.

Most plant species exhibited good tolerance across the assessment days (d0, d8, and d15). Ferns (e.g., *Nephrolepis auriculata* , *Phymatodes scolopendria* ), Asparagaceae species ( *Aspidistra elatior* and its cultivars), and Lamiaceae species (*Scutellaria* spp.) showed particularly high tolerance (Table 1). *k*‐means clustering analysis of the 44 species based on chlorophyll fluorescence further classified them into an immersion‐intolerant group (3 species), a moderately tolerant group (17 species), and a tolerant group (24 species) (Figure 3). Among the tolerant species, 79% were native and 21% were non‐native or horticultural cultivars; in the moderately tolerant group, 76% were native and 24% were non‐native; the immersion‐intolerant group comprised three native *Eupatorium* species.

### 3.2 Chlorophyll Fluorescence (*F*_v_/*F*_m_) of 10 Species Under Cyclical Water Variation

Table 2 summarizes the chlorophyll fluorescence ratio (*F*_v_/*F*_m_) and the proportion of values > 0.7 for 10 plant species under different water stress conditions. Yuan and Dunnett (2018) used a threshold value of 0.7 for *F*_v_/*F*_m_ to determine the tolerance of their test species to water stress at different time periods. Results of this study showed that nine species had *F*_v_/*F*_m_ ratios of > 0.7, ranging from 80% to 100% during the stress period, while only *E. lindleyanum* had experimental data > 0.7 for 66% and 33% of the 7‐ and 14‐day groups, respectively. In addition, the mean *F*_v_/*F*_m_ ratio for all test plants (including experimental and control groups) was 0.805. *A. shimadae* and *R. scabra* had *F*_v_/*F*_m_ ratios greater than the mean value in the 7‐day treatment group, whereas *A. indicus* had a greater ratio in the 14‐day treatment group during the study.

**TABLE 2:** Chlorophyll fluorescence (*F*_v_/*F*_m_) in response to different water stress periods.

Family name	Scientific name	Group	Chlorophyll fluorescence ratio (*F*_v_/*F*_m_)						*F*_v_/*F*_m_ > 0.7 (%)
			W0	W1	D1	W2	D2	W3	
Asteraceae	*Aster indicus*	7‐day treatment	0.820	0.823	0.652	0.818	0.824	0.817	83
		14‐day treatment	0.831	0.833	0.839	0.848	0.841	0.819	100
		7‐day control	0.823	0.826	0.815	0.813	0.816	0.800	
		14‐day control	0.815	0.816	0.800	0.823	0.833	0.839	
Asteraceae	*Aster shimadae*	7‐day treatment	0.840	0.816	0.823	0.829	0.834	0.846	100
		14‐day treatment	0.848	0.784	0.823	0.819	0.842	0.842	100
		7‐day control	0.826	0.817	0.845	0.775	0.831	0.856	
		14‐day control	0.845	0.831	0.856	0.842	0.819	0.854	
Asteraceae	*Eupatorium lindleyanum*	7‐day treatment	0.823	0.782	0.808	0.786	0.519	0.496	66
		14‐day treatment	0.838	0.686	0.809	0.678	0.458	0.370	33
		7‐day control	0.800	0.834	0.818	0.795	0.784	0.821	
		14‐day control	0.818	0.784	0.821	0.828	0.817	0.842	
Campanulaceae	*Lobelia chinensis*	7‐day treatment	0.836	0.831	0.770	0.825	0.832	0.830	100
		14‐day treatment	0.829	0.823	0.835	0.849	0.841	0.796	100
		7‐day control	0.828	0.848	0.829	0.831	0.783	0.792	
		14‐day control	0.829	0.783	0.792	0.836	0.822	0.831	
Caryophyllaceae	*Dianthus seisuimontanus*	7‐day treatment	0.851	0.830	0.851	0.828	0.829	0.798	100
		14‐day treatment	0.847	0.793	0.815	0.799	0.617	0.821	100
		7‐day control	0.852	0.855	0.855	0.838	0.851	0.851	
		14‐day control	0.855	0.851	0.851	0.839	0.831	0.857	
Caryophyllaceae	*Dianthus superbus*	7‐day treatment	0.851	0.830	0.816	0.784	0.769	0.738	100
		14‐day treatment	0.856	0.784	0.825	0.790	0.791	0.805	100
		7‐day control	0.849	0.856	0.861	0.845	0.855	0.846	
		14‐day control	0.861	0.855	0.846	0.843	0.828	0.843	
Convolvulaceae	*Evolvulus alsinoides*	7‐day treatment	0.818	0.815	0.828	0.730	0.834	0.792	100
		14‐day treatment	0.839	0.815	0.821	0.761	0.816	0.824	100
		7‐day control	0.833	0.824	0.840	0.808	0.829	0.821	
		14‐day control	0.840	0.829	0.821	0.823	0.817	0.836	
Euphorbiaceae	*Euphorbia formosana*	7‐day treatment	0.816	0.808	0.842	0.789	0.792	0.812	100
		14‐day treatment	0.847	0.767	0.813	0.781	0.797	0.792	100
		7‐day control	0.828	0.812	0.801	0.761	0.724	0.591	
		14‐day control	0.801	0.724	0.591	0.327	0.332	0.712	
Leguminosae	*Lespedeza cuneata*	7‐day treatment	0.828	0.806	0.790	0.808	0.812	0.783	100
		14‐day treatment	0.838	0.772	0.798	0.772	0.819	0.814	100
		7‐day control	0.834	0.828	0.835	0.792	0.801	0.818	
		14‐day control	0.835	0.801	0.818	0.821	0.803	0.826	
Verbenaceae	*Richardia scabra*	7‐day treatment	0.847	0.835	0.839	0.830	0.814	0.833	100
		14‐day treatment	0.851	0.785	0.825	0.809	0.827	0.833	100
		7‐day control	0.827	0.853	0.814	0.816	0.796	0.831	
		14‐day control	0.814	0.796	0.831	0.831	0.789	0.822	

*Note:* Chlorophyll fluorescence (*F*_v_/*F*_m_) is presented as the mean value. Sample size = 9.

Abbreviations: D1 = first dry period; D2 = second dry period; W0 = before immersion; W1 = first wet period; W2 = second wet period; W3 = third wet period.

**FIGURE 4:**
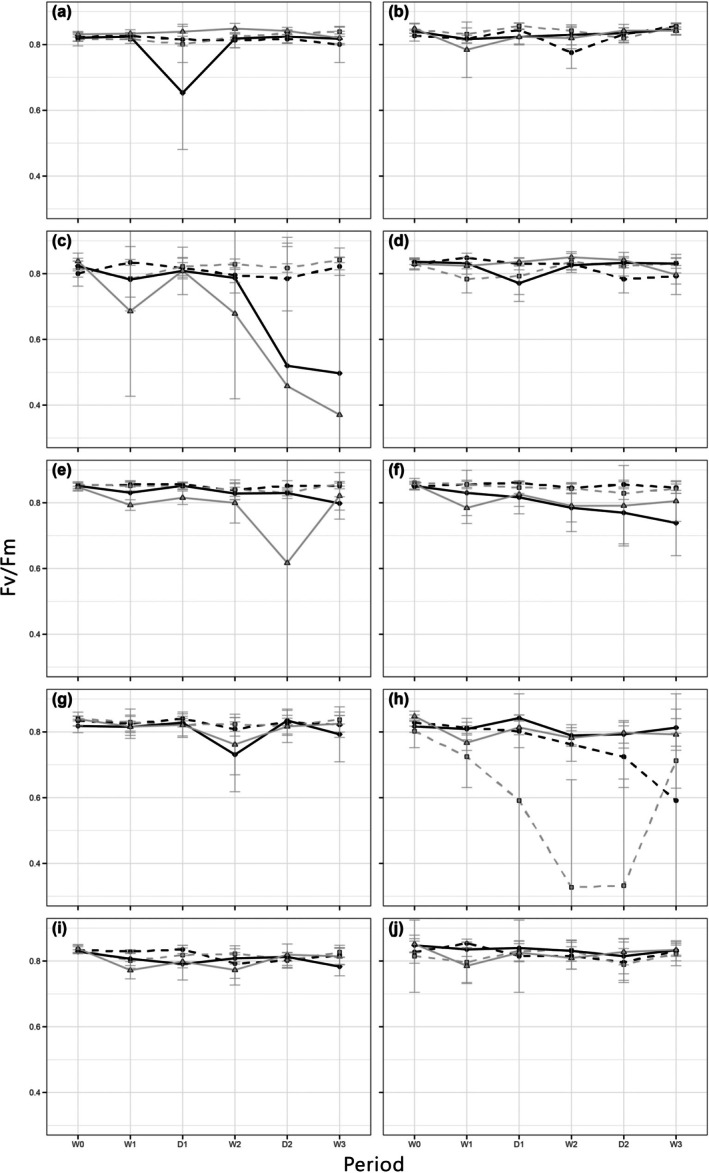
*F*_v_/*F*_m_ values at different time periods. (a) *Aster indicus*, (b) *Aster ahimadae*, (c) *Eupatorium lindleyanum*, (d) *Lobelia chinensis* , (e) *Dianthus seisuimontanus*, (f) *Dianthus superbus* , (g) *Evolvulus alsinoides* , (h) *Euphorbia formosana* , (i) *Lespedeza cuneata* , (j) *Richardia scabra*. And the different lines represent 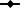: 7‐day treatment, 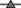: 14‐day treatment, 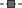: 7‐day control, 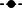: 14‐day control.

Changes in the *F*_v_/*F*_m_ ratio of each species can be observed from the line chart in Figure 4. It was found that the *F*_v_/*F*_m_ value of *E. lindleyanum* showed a significant decreasing trend in the W2 → D2 period of the 7‐day treatment group and in the D1 → W2 period of the 14‐day treatment group, compared to the control group. The pretest results showed that it had a higher tolerance to continuous flooding, but based on the design of this study, it was concluded that *Lindera glauca* had a lower tolerance to periodic flooding and drought stress.

For the control group of *E. formosana* , the *F*_v_/*F*_m_ ratio continued to decrease throughout the experiment. The growth status of control plants showed a gradual withering of aboveground parts with seasonal changes, and after the leaves fell off, new branches and leaves sprouted from the base.

### 3.3 Pairwise *t*‐Tests Were Conducted on Chlorophyll Fluorescence (*F*_v_/*F*_m_) to Determine Tolerance for Different Water Conditions

#### 3.3.1 Changes in Chlorophyll Fluorescence (*F*_v_/*F*_m_) Before and After Stress

According to the nonparametric paired‐sample test results of chlorophyll fluorescence values, the experimental groups of test species at 7 and 14 days showed significant differences in the W0 → W3 period (7W0: 0.833; 7W3: 0.775) (14W0: 0.842; 14W3: 0.772). At the end of the experiment, chlorophyll fluorescence parameters (*F*_v_/*F*_m_) of test species and the control group in the W3 period were compared (7W3: 0.775; 7W3 control: 0.803) (14W3: 0.772; 14W3 control: 0.826), and were not significant in the nonparametric paired‐sample test, indicating that chlorophyll fluorescence parameters (*F*_v_/*F*_m_) of the 7‐ and 14‐day groups under flooding and the control group did not significantly differ (Table 3).

**TABLE 3:** Nonparametric paired‐sample tests conducted on before (W0) and after (W3) the 7‐ and 14‐day waterlogging periods of chlorophyll fluorescence parameters (*F*_v_/*F*_m_).

Cycle	Mean	Standard deviation	*Z*‐score
7W0	0.833	0.01375	−2.803**
7W3	0.775	0.10238	
14W0	0.842	0.0089	−2.803**
14W3	0.772	0.14188	
7W3	0.775	0.10238	−0.866
7W3 control	0.803	0.07729	
14W3	0.772	0.14188	−1.784
14W3 control	0.826	0.04165	

Abbreviations: 7W0 = 7‐day treatment before immersion; 7W3 = 7‐day treatment during the third wet period; 7W3 control = 7‐day control during the third wet period; 14W0 = 14‐day treatment before immersion; 14W3 = 14‐day treatment during the third wet period; 14W3 control = 14‐day control during the third wet period.

**p < 0.01.

#### 3.3.2 Paired‐Sample Nonparametric Test of Tolerance of Chlorophyll Fluorescence (*F*_v_/*F*_m_) for a Single Species Under Different Water Periods

Table 4 presents results of comparing the tolerance of a single species to different water cycle variations. Paired‐sample tests were conducted between the third water immersion (W3) and before immersion (W0) for each species. Regarding changes in chlorophyll fluorescence values for a single species, significant differences were observed for the before and after tests of the 7‐day immersion experiment for *E. lindleyanum* (7W0: 0.823; 7W3: 0.496), *D. seisuimontanus* (7W0: 0.851; 7W3: 0.798), *D. superbus* (7W0: 0.851; 7W3: 0.738), and *L. cuneata* (7W0: 0.816; 7W3: 0.812). However, no differences were observed for the other six species, namely *A. indicus* , *A. shimadae*, *L. chinensis* , *E. formosana* , *R. scabra* , and *E. alsinoides* . For the 14‐day experiment, significant differences were observed for *E. lindleyanum* (14W0: 0.838; 14W3: 0.37), *L. chinensis* (14W0: 0.829; 14W3: 0.796), *D. superbus* (14W0: 0.856; 14W3: 0.805), *E. formosana* (14W0: 0.847; 14W3: 0.792), and *L. cuneata* (14W0: 0.847; 14W3: 0.792). However, no differences were observed for the other species, namely *A. indicus* , *A. shimadae*, *R. scabra* , *E. alsinoides* , and *D. seisuimontanus*. Four species showed no differences in chlorophyll fluorescence values between before and after both the 7‐ and 14‐day stress tests, namely *A. indicus* , *A. shimadae*, *E. alsinoides* , and *R. scabra* .

**TABLE 4:** Nonparametric paired‐sample tests conducted for a single species before (W0) and after (W3) the 7‐ and 14‐day waterlogging periods.

Scientific name	Cycle	Mean	Standard deviation	*Z*‐score
*Aster indicus*	7W0	0.82	0.00971	−0.475
	7W3	0.817	0.01451	
	14W0	0.832	0.00821	−1.601
	14W3	0.819	0.02067	
*Aster shimadae*	7W0	0.84	0.0064	−1.125
	7W3	0.846	0.01782	
	14W0	0.848	0.01387	−0.652
	14W3	0.842	0.01121	
*Eupatorium lindleyanum*	7W0	0.823	0.0147	−2.666**
	7W3	0.496	0.38124	
	14W0	0.838	0.002415	−2.31*
	14W3	0.37	0.44093	
*Lobelia chinensis*	7W0	0.836	0.00819	−0.296
	7W3	0.83	0.02843	
	14W0	0.829	0.01839	−2.668**
	14W3	0.796	0.02815	
*Dianthus seisuimontanus*	7W0	0.851	0.01266	−2.67**
	7W3	0.798	0.02034	
	14W0	0.847	0.0121	−0.356
	14W3	0.821	0.07082	
*Dianthus superbus*	7W0	0.851	0.010145	−2.666**
	7W3	0.738	0.09924	
	14W0	0.856	0.01767	−2.429*
	14W3	0.805	0.06182	
*Evolvulus alsinoides*	7W0	0.818	0.020246	−0.059
	7W3	0.792	0.08337	
	14W0	0.839	0.02113	−1.26
	14W3	0.824	0.02539	
*Euphorbia formosana*	7W0	0.816	0.01786	−0.533
	7W3	0.812	0.0564	
	14W0	0.847	0.01534	−2.192*
	14W3	0.792	0.04803	
*Lespedeza cuneata*	7W0	0.816	0.01786	−2.668**
	7W3	0.812	0.0564	
	14W0	0.847	0.01534	−2.073*
	14W3	0.792	0.04803	
*Richardia scabra*	7W0	0.847	0.02015	−1.791
	7W3	0.833	0.01541	
	14W0	0.851	0.02681	−1.481
	14W3	0.833	0.02003	

Abbreviations: 7W0 = 7‐day treatment before immersion; 7W3 = 7‐day treatment during the third wet period; 14W0 = 14‐day treatment before immersion; 14W3 = 14‐day treatment during the third wet period.

*p < 0.05; **p < 0.01.

## 4 Discussion

### 4.1 Potential Plants for Use in Rain Gardens

The design of rain gardens emphasizes maximizing infiltration and avoiding waterlogging (Dietz and Clausen 2005). Under this premise, the number of days plants remain submerged may vary depending on rainfall duration, which means that plant selection must take into account not only waterlogging tolerance but also climate, soil conditions, and other environmental factors. In this study, plant selection was informed by the expertise of plant ecologists and producers, based on their knowledge of species' natural habitats as well as their moisture tolerance and adaptability in nursery conditions, along with empirical findings that provide preliminary guidelines for practical applications. Among the 44 tested plant species, 24 exhibited no significant visual damage after 15 days of submersion, and their chlorophyll fluorescence parameters showed no notable differences, suggesting that these species could serve as a foundation for expanding plant diversity in rain gardens.

Visual damage assessments further revealed that, except for *Eupatorium hualienense*, which showed injury on the second day, most other plants only began to exhibit moderate stress (level 2 damage, defined as 80%–60% leaf wilting) after 5 days of continuous immersion. Accordingly, if a rain garden is designed to retain surface water for no longer than 3 days, the majority of pretested plants would be suitable candidates. However, considering the highly permeable soils typically used in rain gardens, which can lead to rapid depletion of soil moisture, further research is still required to evaluate the drought tolerance and recovery capacity of these species in order to confirm their long‐term suitability.

### 4.2 Plant Selection for Rain Gardens

Nonparametric paired‐sample tests indicated that during the experiment, four species, *A. indicus* , *A. shimadae*, *E. alsinoides* , and *R. scabra* , showed no significant differences in tolerance to fluctuating water regimes (these species were categorized as immersion‐tolerant in the pretest, and no differences were observed before and after the 7‐ or 14‐day immersion treatments). These species, originally classified as immersion‐tolerant in the pretest, also maintained consistent performance before and after exposure to different water regimes, suggesting their potential for future use in rain gardens.

In contrast, six species, *E. lindleyanum*, *L. chinensis* , *D. seisuimontanus*, *D. superbus* , *E. formosana* , and *L. cuneata* , showed differences in *F*_v_/*F*_m_ values before and after the 7‐ or 14‐day immersion treatments. The results indicate that although these species can tolerate prolonged submergence, such stress may reduce their capacity to cope with drought under varying water regimes.

With respect to the use of native and non‐native species, the pretest results showed that all three tolerance groups included both native, non‐native, and horticultural cultivars. This indicates that superior performance is not strictly limited to native species, which is consistent with the perspective proposed by Bortolini and Zanin (2019) that horticultural cultivars may also be suitable for rain gardens. However, it is noteworthy that within the immersion‐tolerant group, 79% of the species identified were native. This suggests that while non‐native or horticultural species can contribute to rain garden plantings, native species may offer a greater likelihood of adaptation and resilience under fluctuating water regimes, reinforcing their value as priority candidates for selection.

Nelson et al. (2018) tested Cyperaceae species, including wetland, amphibious, and terrestrial plants, and found that terrestrial and amphibious species had higher stress tolerance than wetland species. They suggested using nonaquatic sedges in rain gardens. However, in tropical regions like Taiwan, many Cyperaceae species (e.g., *Cyperus rotundus* , *Kyllinga brevifolia* , *Cyperus iria* ) are considered weeds and are often not favored by the public (Hsu and Chiang 2000), indicating that species selection should consider local ecological conditions and cultural perceptions. Similarly, Eben et al. (2024) found that over 70% of drought‐tolerant perennials maintained normal *F*_v_/*F*_m_ values (~0.74–0.85) and low mortality under combined flooding, drought, and road salt stress, suggesting that drought‐hardy species may also tolerate immersion and salinity.

Compared to our study, similar patterns were observed: *L. chinensis* , a wetland species native to moist habitats, showed significant differences in chlorophyll fluorescence after the 14‐day immersion treatment. This indicates that wetland plants may not be a priority choice for rain garden species selection. Instead, species that naturally tolerate drought or fluctuating soil moisture, such as coastal plants, species from limestone or karst terrains, and riparian plants should be prioritized.

In addition, future selection of suitable rain garden species could also refer to seed‐based screening methods. For example, Chen et al. (2008) mentioned that in the breeding process of immersion‐tolerant plants, *Glycine max* can use pre‐flooding treatment during seed germination as a screening method. Seed germination and physiological performance of different varieties of *B. chinensis* also showed a high correlation with immersion tolerance, and post‐flooding germination rates can serve as an early screening indicator. Therefore, such methods may be applied in the future selection of flood‐tolerant plant species.

### 4.3 Can the Tolerance to Water Stress Be Determined Based on the Plant's Habitat?

Plant guides in Taiwan often describe the ecological environments and cultivation requirements of species, such as *E. formosana* in sandy coastal areas, *L. chinensis* in lowland wetlands, and the need for good drainage in *A. indicus* (Zhang et al. 2018a, 2018b; Xue 2000). However, experimental results showed that these species exhibited no significant differences in *F*_v_/*F*_m_ values during a 7‐day immersion treatment simulating periodic water fluctuation stress, suggesting that natural habitats or cultivation conditions cannot reliably predict tolerance to water stress. Similar findings were reported by Yuan and Dunnett (2018), where only four out of 15 species listed in a rain garden guide demonstrated higher tolerance to fluctuating water regimes. Together, these results indicate that habitat descriptions alone are insufficient to determine species' tolerance to water stress, underscoring the need for empirical testing and the development of a plant list tailored for rain gardens.

### 4.4 Method for Assessing Plants Tolerance to Water Stress

Plants are primary greening materials in open spaces, and their visual aesthetics can affect viewer perception. In the preliminary assessment of flood tolerance, both visual and physiological responses were evaluated, revealing that 54% of species showed no change before and after submersion. Therefore, in the main experiment, tolerance to different water cycles was assessed using the *F*_v_/*F*_m_ ratio. Flood stress can induce ATP deficiency, disrupt cell membrane redox potential, increase membrane permeability, and lead to ion and metabolite leakage, ultimately reducing *F*_v_/*F*_m_ (Chen et al. 2008; Yuan and Dunnett 2018). Liu (2012) also suggested that chlorophyll fluorescence parameters could be used to evaluate crops' resistance to adverse environmental stress, supporting their use as a precise physiological indicator in stress tolerance assessments.

In this study, most species exhibited no significant *F*_v_/*F*_m_ changes after stress cycles, likely due to the preliminary selection of more submersion‐tolerant species. Notably, *F*_v_/*F*_m_ changes did not always correspond to visible symptoms such as leaf yellowing or wilting (Hsu 2012). However, even tolerant species may experience reduced root and shoot growth (Yuan and Dunnett 2018), consistent with observations in Cyperaceae under drought stress (Nelson et al. 2018). Visual comparisons in this study showed measurable changes in height and leaf size for species such as *E. lindleyanum* and *E. formosana* .

These results suggest that visual assessment can serve as an initial screening for stress tolerance, while chlorophyll fluorescence provides a more precise tool for distinguishing species with similar tolerance. The physiological mechanisms underlying this dual tolerance are further supported by the theoretical framework proposed by Chen et al. (2023), which integrates the similar ecophysiological responses plants exhibit under both drought and waterlogging stress—such as stomatal closure, inhibited gas exchange, and photoinhibition reflected in declining *F*_v_/*F*_m_. Combined, these approaches allow early detection of physiological responses and provide a comprehensive evaluation.

### 4.5 Factors Affecting Chlorophyll Fluorescence (*F*_v_/*F*_m_)

After subjecting plants to stress tests, paired‐sample tests were conducted to examine changes in chlorophyll fluorescence (*F*_v_/*F*_m_) among species with different water tolerance levels. Results showed that during the W0 → W3 period, significant differences in *F*_v_/*F*_m_ were observed among plant species in nonparametric paired‐sample tests.

Two possible explanations were identified. First, the root growth response of plants to short‐term changes in water availability was less pronounced (Glenz et al. 2006; Nelson et al. 2018). In the main experiment, the duration of wet–dry cycles was relatively short, giving plant roots insufficient time to respond, which led to a decrease in *F*_v_/*F*_m_ for some species, such as *E. lindleyanum* and *D. superbus* . Second, seasonal temperature changes may have contributed. Weng et al. (2011) reported that *F*_v_/*F*_m_ values measured before dawn under low‐temperature stress in winter decrease with declining seasonal temperatures. In this study, measurements were conducted from November 8, 2021, to January 24, 2022, corresponding to winter in Chiayi, Taiwan. The monthly average temperatures recorded at the Chiayi weather station were 22.2°C in November, 18.4°C in December, and 17.7°C in January. Therefore, the observed changes in *F*_v_/*F*_m_ before and after the stress treatment may also have been influenced by decreasing environmental temperatures.

To improve the applicability of plant lists, it should be noted that plant growth may be affected by factors such as temperature and rainfall. In future experiments of this type, controlling temperature or the number of consecutive days of immersion could help establish plant lists suitable for different regional characteristics.

## 5 Conclusion

The development of a plant list suitable for rain gardens is urgently needed in the context of climate change. In this study, 44 species were selected through interviews and analyses of native habitats, and their tolerance to water stress was evaluated using both visual assessments and chlorophyll fluorescence (*F*_v_/*F*_m_). Four native Taiwanese species, *A. indicus* , *A. shimadae*, *E. alsinoides* , and *R. scabra* , were identified as suitable candidates for rain garden applications. Furthermore, the results demonstrate that habitat descriptions alone are insufficient to determine species' tolerance to water stress, underscoring the need for empirical testing and the establishment of a plant list specifically tailored for rain gardens. While both native and non‐native species showed varying levels of tolerance, the predominance of native species among immersion‐tolerant plants highlights their greater potential for adaptation under fluctuating water regimes. In terms of assessment methods, *F*_v_/*F*_m_ changes did not always correspond to visible symptoms such as leaf yellowing or wilting. Therefore, combining visual evaluation with chlorophyll fluorescence allows for the early detection of physiological responses and provides a more comprehensive assessment of plant tolerance.

## Conflicts of Interest

The authors declare no conflicts of interest.

## Data Availability

The data supporting the findings of this study are included within the article. No additional data are available.
